# (2-Methyl-1-phenyl­sulfonyl-1*H*-indol-3-yl)phenyl­methyl acetate

**DOI:** 10.1107/S1600536809044365

**Published:** 2009-10-28

**Authors:** B. Saravanan, V. Dhayalan, A. K. Mohanakrishnan, G. Chakkaravarthi, V. Manivannan

**Affiliations:** a Department of Research and Development, PRIST University, Vallam, Thanjavur 613 403, Tamil Nadu, India; b Department of Organic Chemistry, University of Madras, Guindy Campus, Chennai 600 025, India; c Department of Physics, CPCL Polytechnic College, Chennai 600 068, India

## Abstract

In the title compound, C_24_H_21_NO_4_S, the indole ring system makes dihedral angles of 77.8 (1) and 85.4 (1)°, respectively, with the S- and C-bound phenyl rings. The mol­ecular structure is stabilized by a weak intra­molecular C—H⋯O hydrogen bond. In the crystal, a weak inter­molecular C—H⋯O hydrogen bond and a C—H⋯π inter­action are also observed.

## Related literature

For the biological activity of indole derivatives, see: Chai *et al.* (2006 ▶); Olgen & Coban (2003 ▶). For related structures, see: Chakkaravarthi *et al.* (2007 ▶, 2008 ▶).
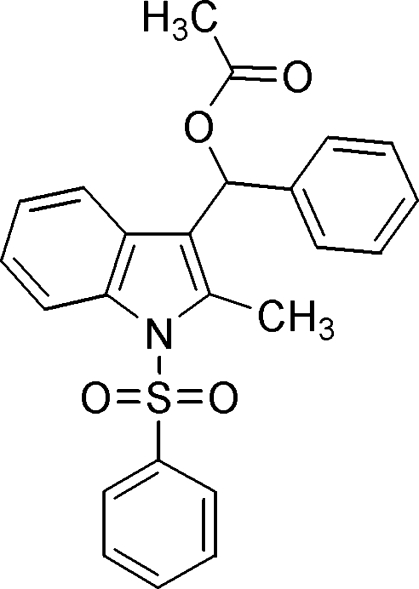


## Experimental

### 

#### Crystal data

C_24_H_21_NO_4_S
*M* *_r_* = 419.48
Monoclinic, 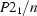

*a* = 14.3655 (6) Å
*b* = 8.3432 (4) Å
*c* = 18.6261 (8) Å
β = 108.086 (2)°
*V* = 2122.12 (16) Å^3^
*Z* = 4
Mo *K*α radiation
μ = 0.18 mm^−1^
*T* = 295 K
0.28 × 0.24 × 0.18 mm

#### Data collection

Bruker Kappa APEXII diffractometer
Absorption correction: multi-scan (**SADABS**; Sheldrick, 1996 ▶) *T*_min_ = 0.951, *T*_max_ = 0.968
23327 measured reflections
4712 independent reflections
3030 reflections with *I* > 2σ(*I*)
*R*_int_ = 0.043

#### Refinement

*R*[*F* ^2^ > 2σ(*F* ^2^)] = 0.047
*wR*(*F* ^2^) = 0.146
*S* = 1.01
4712 reflections
273 parameters
2 restraints
H-atom parameters constrained
Δρ_max_ = 0.26 e Å^−3^
Δρ_min_ = −0.35 e Å^−3^

### 

Data collection: *APEX2* (Bruker, 2004 ▶); cell refinement: *SAINT* (Bruker, 2004 ▶); data reduction: *SAINT*; program(s) used to solve structure: *SHELXS97* (Sheldrick, 2008 ▶); program(s) used to refine structure: *SHELXL97* (Sheldrick, 2008 ▶); molecular graphics: *PLATON* (Spek, 2009 ▶); software used to prepare material for publication: *SHELXL97*.

## Supplementary Material

Crystal structure: contains datablocks global, I. DOI: 10.1107/S1600536809044365/is2478sup1.cif

Structure factors: contains datablocks I. DOI: 10.1107/S1600536809044365/is2478Isup2.hkl

Additional supplementary materials:  crystallographic information; 3D view; checkCIF report

## Figures and Tables

**Table 1:** Hydrogen-bond geometry (Å, °)

*D*—H⋯*A*	*D*—H	H⋯*A*	*D*⋯*A*	*D*—H⋯*A*
C13—H13⋯O1	0.93	2.39	2.977 (4)	121
C24—H24*B*⋯O2^i^	0.96	2.58	3.429 (4)	147
C15—H15*A*⋯*Cg*1^ii^	0.96	2.97	3.590 (3)	124

Symmetry codes: (i) 
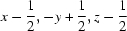
; (ii) 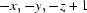. *Cg*1 is the centroid of the C17–C22 ring.

**Fig. 1.**
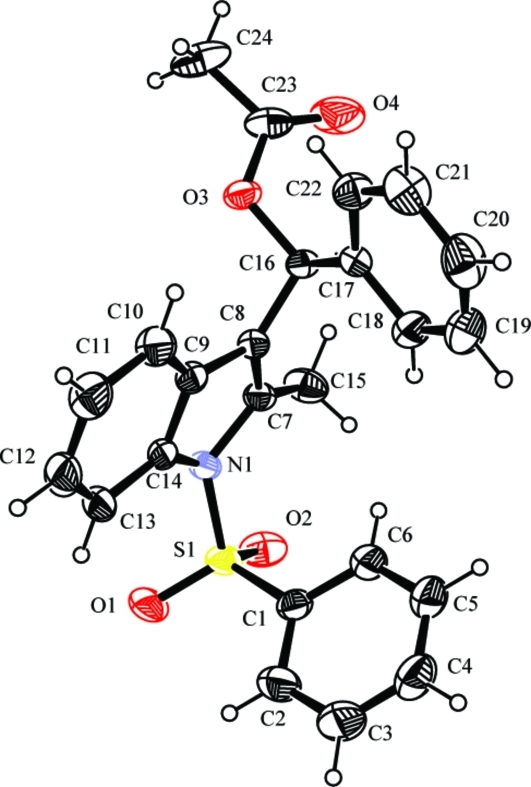
The molecular structure of (I), with atom labels and 30% probability displacement ellipsoids for non-H atoms.

**Fig. 2.**
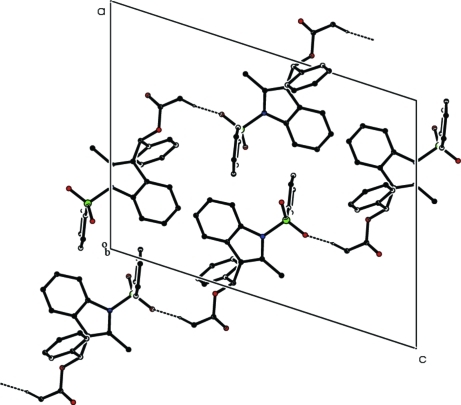
The packing of (I), viewed down the b axis. Hydrogen bonds are shown as dashed lines. H atoms not involved in hydrogen bonding have been omitted.

## supplementary crystallographic information

### Comment

In continuation of our studies of indole derivatives, which are known to exhibit antihepatitis B virus (Chai *et al.*, 2006) and anti-oxidant activity (Olgen & Coban, 2003), we report the crystal structure of the title compound, (I). The bond lengths and bond angles of the title compound are agree with the reported similar structures (Chakkaravarthi *et al.*, 2007,2008).

The phenyl rings C1—C6 and C17—C22 make dihedral angles of 77.8 (1) and 85.4 (1)°, respectively, with the indole ring system. The two phenyl rings are inclined at an angle of 62.2 (1)° with respect to each other. The torsion angles C7—N1—S1—O2 and C14—N1—S1—O1 [-36.8 (2) and 51.1 (2)°, respectively] indicate a *syn* conformation of the sulfonyl moiety. The sum of the bond angles around N1 [351.2 (2)°] indicates that N1 is *sp*^2^-hybridized.

The molecular structure is controlled by a weak intramolecular C—H···O hydrogen bond and the crystal packing of (I) (Fig. 2) is through weak intermolecular C—H···O hydrogen bonds and C—H···π (Table 1) interactions.

### Experimental

To a solution of 1-phenylsulfonyl-(2-methyl-1*H*-indol-3-yl) (phenyl)methanol (0.5 g, 1.32 mmol) in dry DCM (20 ml) acetic anhydride (0.27 g, 2.64 mmol) and pyridine (0.2 g, 2.52 mmol) were added. It was then stirred at room temperature for 7 h under N_2_ atmosphere. The reaction mixture was poured over crushed ice (100 g) containing 2 ml of Conc. HCl, extracted with CHCl_3_ (3 × 10 ml) and dried (Na_2_SO_4_). Removal of solvent followed by recrystallization from CDCl_3_ afforded the compound as crystals.

### Refinement

H atoms were positioned geometrically and refined using riding model with C—H = 0.93 Å and *U*_iso_(H) = 1.2*U*_eq_(C) for aromatic C—H, C—H = 0.98 Å and *U*_iso_(H) = 1.2*U*_eq_(C) for C—H, and C—H = 0.96 Å and *U*_iso_(H) = 1.5*U*_eq_(C) for methyl. The components of the anisotropic displacement parameters in direction of the bond of S1and O2; C3 and C4 were restrained to be equal within an effective standard deviation of 0.001 using the DELU command in *SHELXL* (Sheldrick, 2008).

### Crystal data

**Table:**

C_24_H_21_NO_4_S	*F*(000) = 880
*M**_r_* = 419.48	*D*_x_ = 1.313 Mg m^−^^3^
Monoclinic, *P*2_1_/*n*	Mo *K*α radiation, λ = 0.71073 Å
Hall symbol: -P 2yn	Cell parameters from 6715 reflections
*a* = 14.3655 (6) Å	θ = 2.3–25.3°
*b* = 8.3432 (4) Å	µ = 0.18 mm^−^^1^
*c* = 18.6261 (8) Å	*T* = 295 K
β = 108.086 (2)°	Block, colourless
*V* = 2122.12 (16) Å^3^	0.28 × 0.24 × 0.18 mm
*Z* = 4	

### Data collection

**Table:**

Bruker Kappa APEXII diffractometer	4712 independent reflections
Radiation source: fine-focus sealed tube	3030 reflections with *I* > 2σ(*I*)
graphite	*R*_int_ = 0.043
ω and φ scans	θ_max_ = 27.4°, θ_min_ = 2.2°
Absorption correction: multi-scan (*SADABS*; Sheldrick, 1996)	*h* = −18→18
*T*_min_ = 0.951, *T*_max_ = 0.968	*k* = −9→10
23327 measured reflections	*l* = −23→24

### Refinement

**Table:**

Refinement on *F*^2^	Primary atom site location: structure-invariant direct methods
Least-squares matrix: full	Secondary atom site location: difference Fourier map
*R*[*F*^2^ > 2σ(*F*^2^)] = 0.047	Hydrogen site location: inferred from neighbouring sites
*wR*(*F*^2^) = 0.146	H-atom parameters constrained
*S* = 1.01	*w* = 1/[σ^2^(*F*_o_^2^) + (0.0657*P*)^2^ + 0.817*P*] where *P* = (*F*_o_^2^ + 2*F*_c_^2^)/3
4712 reflections	(Δ/σ)_max_ < 0.001
273 parameters	Δρ_max_ = 0.26 e Å^−^^3^
2 restraints	Δρ_min_ = −0.35 e Å^−^^3^

### Fractional atomic coordinates and isotropic or equivalent isotropic displacement parameters (Å^2^)

**Table:**

	*x*	*y*	*z*	*U*_iso_*/*U*_eq_	
C1	0.39968 (15)	0.2073 (3)	0.58199 (12)	0.0515 (5)	
C2	0.49717 (19)	0.1911 (4)	0.58978 (19)	0.0807 (8)	
H2	0.5361	0.2802	0.5897	0.097*	
C3	0.5362 (2)	0.0363 (4)	0.5978 (2)	0.0976 (10)	
H3	0.6024	0.0216	0.6039	0.117*	
C4	0.4786 (2)	−0.0927 (4)	0.59684 (17)	0.0836 (8)	
H4	0.5056	−0.1949	0.6014	0.100*	
C5	0.3827 (2)	−0.0755 (3)	0.58934 (16)	0.0741 (7)	
H5	0.3439	−0.1652	0.5885	0.089*	
C6	0.34286 (18)	0.0747 (3)	0.58297 (14)	0.0645 (7)	
H6	0.2772	0.0875	0.5793	0.077*	
C7	0.15867 (14)	0.3154 (3)	0.49542 (11)	0.0430 (5)	
C8	0.11839 (14)	0.2508 (2)	0.42695 (11)	0.0416 (5)	
C9	0.18128 (14)	0.2795 (3)	0.38139 (11)	0.0448 (5)	
C10	0.17326 (19)	0.2446 (3)	0.30680 (13)	0.0623 (6)	
H10	0.1193	0.1894	0.2762	0.075*	
C11	0.2471 (2)	0.2937 (4)	0.27933 (16)	0.0780 (8)	
H11	0.2429	0.2711	0.2295	0.094*	
C12	0.3271 (2)	0.3760 (4)	0.32454 (18)	0.0768 (8)	
H12	0.3758	0.4084	0.3044	0.092*	
C13	0.33684 (17)	0.4113 (3)	0.39800 (16)	0.0624 (7)	
H13	0.3913	0.4662	0.4283	0.075*	
C14	0.26243 (14)	0.3622 (2)	0.42576 (12)	0.0450 (5)	
C15	0.11753 (18)	0.3291 (4)	0.55899 (14)	0.0671 (7)	
H15A	0.0505	0.2942	0.5427	0.101*	
H15B	0.1207	0.4388	0.5752	0.101*	
H15C	0.1547	0.2633	0.6002	0.101*	
C16	0.02422 (14)	0.1601 (3)	0.40188 (11)	0.0441 (5)	
H16	−0.0023	0.1524	0.4443	0.053*	
C17	0.03687 (14)	−0.0071 (3)	0.37520 (12)	0.0462 (5)	
C18	0.10644 (19)	−0.1056 (3)	0.42217 (16)	0.0698 (7)	
H18	0.1445	−0.0681	0.4691	0.084*	
C19	0.1201 (2)	−0.2590 (4)	0.4002 (2)	0.0888 (10)	
H19	0.1675	−0.3240	0.4324	0.107*	
C20	0.0652 (3)	−0.3164 (4)	0.3322 (2)	0.0871 (9)	
H20	0.0751	−0.4199	0.3175	0.105*	
C21	−0.0047 (2)	−0.2208 (4)	0.28573 (17)	0.0763 (8)	
H21	−0.0434	−0.2600	0.2393	0.092*	
C22	−0.01860 (17)	−0.0670 (3)	0.30661 (13)	0.0570 (6)	
H22	−0.0661	−0.0028	0.2740	0.068*	
C23	−0.13810 (17)	0.2461 (4)	0.33675 (15)	0.0638 (7)	
C24	−0.1970 (2)	0.3458 (4)	0.27301 (16)	0.0905 (10)	
H24A	−0.2652	0.3353	0.2684	0.136*	
H24B	−0.1863	0.3107	0.2271	0.136*	
H24C	−0.1777	0.4559	0.2822	0.136*	
N1	0.24997 (11)	0.3880 (2)	0.49744 (10)	0.0460 (4)	
O1	0.41254 (12)	0.5105 (2)	0.55926 (11)	0.0776 (6)	
O2	0.31180 (12)	0.4240 (2)	0.63643 (10)	0.0722 (5)	
O3	−0.04306 (10)	0.25213 (18)	0.34207 (8)	0.0521 (4)	
O4	−0.16788 (13)	0.1681 (4)	0.37807 (14)	0.1077 (8)	
S1	0.34717 (4)	0.39792 (7)	0.57453 (3)	0.0550 (2)	

### Atomic displacement parameters (Å^2^)

**Table:**

	*U*^11^	*U*^22^	*U*^33^	*U*^12^	*U*^13^	*U*^23^
C1	0.0450 (12)	0.0497 (13)	0.0509 (12)	0.0019 (10)	0.0020 (9)	0.0002 (10)
C2	0.0482 (14)	0.0716 (19)	0.117 (2)	0.0010 (13)	0.0178 (15)	0.0069 (16)
C3	0.0590 (17)	0.089 (2)	0.143 (3)	0.0230 (13)	0.0295 (18)	0.018 (2)
C4	0.091 (2)	0.0648 (18)	0.093 (2)	0.0246 (12)	0.0245 (17)	0.0178 (15)
C5	0.0770 (18)	0.0534 (16)	0.0851 (19)	0.0061 (13)	0.0152 (15)	0.0120 (13)
C6	0.0546 (14)	0.0528 (15)	0.0786 (17)	0.0003 (11)	0.0098 (12)	0.0062 (12)
C7	0.0348 (10)	0.0437 (12)	0.0481 (11)	0.0009 (8)	0.0095 (8)	−0.0012 (9)
C8	0.0350 (10)	0.0425 (11)	0.0461 (11)	0.0017 (8)	0.0110 (8)	0.0030 (8)
C9	0.0403 (11)	0.0432 (12)	0.0510 (12)	0.0054 (9)	0.0142 (9)	0.0070 (9)
C10	0.0609 (14)	0.0761 (18)	0.0529 (13)	0.0012 (12)	0.0222 (11)	0.0023 (12)
C11	0.0809 (19)	0.101 (2)	0.0631 (15)	0.0118 (17)	0.0387 (15)	0.0146 (15)
C12	0.0630 (17)	0.089 (2)	0.093 (2)	0.0123 (15)	0.0453 (16)	0.0327 (17)
C13	0.0435 (12)	0.0586 (16)	0.0878 (18)	0.0013 (10)	0.0243 (12)	0.0209 (13)
C14	0.0359 (10)	0.0392 (12)	0.0596 (13)	0.0060 (8)	0.0146 (9)	0.0117 (9)
C15	0.0537 (14)	0.091 (2)	0.0574 (14)	−0.0115 (13)	0.0185 (11)	−0.0183 (13)
C16	0.0354 (10)	0.0514 (13)	0.0408 (10)	−0.0007 (9)	0.0050 (8)	0.0027 (9)
C17	0.0374 (10)	0.0471 (13)	0.0515 (12)	−0.0045 (9)	0.0102 (9)	0.0053 (9)
C18	0.0609 (15)	0.0552 (16)	0.0789 (17)	0.0024 (12)	0.0009 (13)	0.0109 (13)
C19	0.082 (2)	0.0535 (18)	0.121 (3)	0.0137 (15)	0.0171 (19)	0.0200 (17)
C20	0.103 (2)	0.0510 (17)	0.120 (3)	0.0054 (17)	0.052 (2)	−0.0065 (17)
C21	0.089 (2)	0.0676 (19)	0.0777 (18)	−0.0066 (16)	0.0334 (16)	−0.0204 (15)
C22	0.0571 (13)	0.0588 (15)	0.0527 (13)	0.0017 (11)	0.0135 (11)	−0.0047 (11)
C23	0.0402 (12)	0.0856 (19)	0.0601 (14)	0.0121 (12)	0.0077 (11)	−0.0171 (13)
C24	0.0653 (17)	0.114 (3)	0.0726 (17)	0.0443 (17)	−0.0071 (14)	−0.0203 (16)
N1	0.0352 (9)	0.0430 (10)	0.0556 (10)	−0.0032 (7)	0.0082 (7)	−0.0001 (8)
O1	0.0533 (10)	0.0505 (11)	0.1134 (15)	−0.0194 (8)	0.0033 (9)	−0.0008 (10)
O2	0.0649 (10)	0.0761 (12)	0.0635 (9)	0.0035 (9)	0.0021 (7)	−0.0289 (9)
O3	0.0376 (8)	0.0569 (10)	0.0549 (9)	0.0070 (6)	0.0041 (6)	0.0029 (7)
O4	0.0438 (10)	0.180 (3)	0.1018 (16)	0.0050 (13)	0.0256 (11)	0.0205 (17)
S1	0.0428 (3)	0.0439 (3)	0.0671 (4)	−0.0055 (2)	0.0009 (2)	−0.0092 (3)

### Geometric parameters (Å, °)

**Table:**

C1—C2	1.369 (3)	C14—N1	1.418 (3)
C1—C6	1.378 (3)	C15—H15A	0.9600
C1—S1	1.747 (2)	C15—H15B	0.9600
C2—C3	1.398 (4)	C15—H15C	0.9600
C2—H2	0.9300	C16—O3	1.448 (2)
C3—C4	1.354 (5)	C16—C17	1.510 (3)
C3—H3	0.9300	C16—H16	0.9800
C4—C5	1.349 (4)	C17—C22	1.373 (3)
C4—H4	0.9300	C17—C18	1.377 (3)
C5—C6	1.368 (4)	C18—C19	1.376 (4)
C5—H5	0.9300	C18—H18	0.9300
C6—H6	0.9300	C19—C20	1.354 (5)
C7—C8	1.339 (3)	C19—H19	0.9300
C7—N1	1.434 (3)	C20—C21	1.362 (4)
C7—C15	1.483 (3)	C20—H20	0.9300
C8—C9	1.438 (3)	C21—C22	1.373 (4)
C8—C16	1.493 (3)	C21—H21	0.9300
C9—C14	1.386 (3)	C22—H22	0.9300
C9—C10	1.389 (3)	C23—O4	1.184 (3)
C10—C11	1.375 (4)	C23—O3	1.339 (3)
C10—H10	0.9300	C23—C24	1.481 (4)
C11—C12	1.379 (4)	C24—H24A	0.9600
C11—H11	0.9300	C24—H24B	0.9600
C12—C13	1.364 (4)	C24—H24C	0.9600
C12—H12	0.9300	N1—S1	1.6653 (17)
C13—C14	1.386 (3)	O1—S1	1.4181 (18)
C13—H13	0.9300	O2—S1	1.4137 (19)
			
C2—C1—C6	120.7 (2)	H15A—C15—H15C	109.5
C2—C1—S1	120.0 (2)	H15B—C15—H15C	109.5
C6—C1—S1	119.25 (18)	O3—C16—C8	107.17 (17)
C1—C2—C3	117.7 (3)	O3—C16—C17	110.88 (16)
C1—C2—H2	121.1	C8—C16—C17	112.41 (17)
C3—C2—H2	121.1	O3—C16—H16	108.8
C4—C3—C2	120.7 (3)	C8—C16—H16	108.8
C4—C3—H3	119.7	C17—C16—H16	108.8
C2—C3—H3	119.7	C22—C17—C18	118.1 (2)
C5—C4—C3	121.1 (3)	C22—C17—C16	123.24 (19)
C5—C4—H4	119.4	C18—C17—C16	118.7 (2)
C3—C4—H4	119.4	C19—C18—C17	120.5 (3)
C4—C5—C6	119.5 (3)	C19—C18—H18	119.7
C4—C5—H5	120.2	C17—C18—H18	119.7
C6—C5—H5	120.2	C20—C19—C18	120.8 (3)
C5—C6—C1	120.2 (2)	C20—C19—H19	119.6
C5—C6—H6	119.9	C18—C19—H19	119.6
C1—C6—H6	119.9	C19—C20—C21	119.2 (3)
C8—C7—N1	108.42 (17)	C19—C20—H20	120.4
C8—C7—C15	129.05 (19)	C21—C20—H20	120.4
N1—C7—C15	122.29 (18)	C20—C21—C22	120.7 (3)
C7—C8—C9	109.27 (18)	C20—C21—H21	119.6
C7—C8—C16	125.70 (18)	C22—C21—H21	119.6
C9—C8—C16	125.02 (18)	C21—C22—C17	120.7 (2)
C14—C9—C10	119.6 (2)	C21—C22—H22	119.6
C14—C9—C8	107.29 (18)	C17—C22—H22	119.6
C10—C9—C8	133.1 (2)	O4—C23—O3	122.6 (2)
C11—C10—C9	118.4 (3)	O4—C23—C24	126.7 (3)
C11—C10—H10	120.8	O3—C23—C24	110.7 (3)
C9—C10—H10	120.8	C23—C24—H24A	109.5
C10—C11—C12	121.0 (3)	C23—C24—H24B	109.5
C10—C11—H11	119.5	H24A—C24—H24B	109.5
C12—C11—H11	119.5	C23—C24—H24C	109.5
C13—C12—C11	121.8 (2)	H24A—C24—H24C	109.5
C13—C12—H12	119.1	H24B—C24—H24C	109.5
C11—C12—H12	119.1	C14—N1—C7	107.05 (16)
C12—C13—C14	117.4 (2)	C14—N1—S1	120.16 (13)
C12—C13—H13	121.3	C7—N1—S1	123.97 (14)
C14—C13—H13	121.3	C23—O3—C16	117.29 (19)
C9—C14—C13	121.9 (2)	O2—S1—O1	119.30 (12)
C9—C14—N1	107.96 (17)	O2—S1—N1	107.07 (10)
C13—C14—N1	130.1 (2)	O1—S1—N1	106.87 (10)
C7—C15—H15A	109.5	O2—S1—C1	109.33 (11)
C7—C15—H15B	109.5	O1—S1—C1	108.81 (11)
H15A—C15—H15B	109.5	N1—S1—C1	104.41 (9)
C7—C15—H15C	109.5		
			
C6—C1—C2—C3	0.7 (4)	C8—C16—C17—C18	53.1 (3)
S1—C1—C2—C3	178.1 (2)	C22—C17—C18—C19	0.6 (4)
C1—C2—C3—C4	0.8 (5)	C16—C17—C18—C19	179.7 (2)
C2—C3—C4—C5	−1.1 (5)	C17—C18—C19—C20	−0.3 (5)
C3—C4—C5—C6	−0.3 (5)	C18—C19—C20—C21	−0.5 (5)
C4—C5—C6—C1	1.9 (4)	C19—C20—C21—C22	1.1 (5)
C2—C1—C6—C5	−2.1 (4)	C20—C21—C22—C17	−0.8 (4)
S1—C1—C6—C5	−179.5 (2)	C18—C17—C22—C21	−0.1 (3)
N1—C7—C8—C9	−0.4 (2)	C16—C17—C22—C21	−179.1 (2)
C15—C7—C8—C9	174.1 (2)	C9—C14—N1—C7	0.9 (2)
N1—C7—C8—C16	178.36 (18)	C13—C14—N1—C7	178.2 (2)
C15—C7—C8—C16	−7.1 (4)	C9—C14—N1—S1	149.55 (15)
C7—C8—C9—C14	0.9 (2)	C13—C14—N1—S1	−33.1 (3)
C16—C8—C9—C14	−177.81 (19)	C8—C7—N1—C14	−0.3 (2)
C7—C8—C9—C10	−177.2 (2)	C15—C7—N1—C14	−175.3 (2)
C16—C8—C9—C10	4.0 (4)	C8—C7—N1—S1	−147.47 (16)
C14—C9—C10—C11	0.1 (4)	C15—C7—N1—S1	37.6 (3)
C8—C9—C10—C11	178.1 (2)	O4—C23—O3—C16	−0.8 (4)
C9—C10—C11—C12	−0.2 (4)	C24—C23—O3—C16	−179.9 (2)
C10—C11—C12—C13	0.4 (5)	C8—C16—O3—C23	−146.89 (19)
C11—C12—C13—C14	−0.5 (4)	C17—C16—O3—C23	90.1 (2)
C10—C9—C14—C13	−0.3 (3)	C14—N1—S1—O2	−179.98 (16)
C8—C9—C14—C13	−178.72 (19)	C7—N1—S1—O2	−36.81 (19)
C10—C9—C14—N1	177.35 (19)	C14—N1—S1—O1	51.11 (18)
C8—C9—C14—N1	−1.1 (2)	C7—N1—S1—O1	−165.73 (17)
C12—C13—C14—C9	0.4 (3)	C14—N1—S1—C1	−64.10 (17)
C12—C13—C14—N1	−176.6 (2)	C7—N1—S1—C1	79.06 (18)
C7—C8—C16—O3	115.6 (2)	C2—C1—S1—O2	−117.5 (2)
C9—C8—C16—O3	−65.9 (2)	C6—C1—S1—O2	59.9 (2)
C7—C8—C16—C17	−122.4 (2)	C2—C1—S1—O1	14.3 (3)
C9—C8—C16—C17	56.2 (3)	C6—C1—S1—O1	−168.25 (19)
O3—C16—C17—C22	−7.9 (3)	C2—C1—S1—N1	128.2 (2)
C8—C16—C17—C22	−127.9 (2)	C6—C1—S1—N1	−54.4 (2)
O3—C16—C17—C18	173.0 (2)		

### Hydrogen-bond geometry (Å, °)

**Table:**

*D*—H···*A*	*D*—H	H···*A*	*D*···*A*	*D*—H···*A*
C13—H13···O1	0.93	2.39	2.977 (4)	121
C24—H24B···O2^i^	0.96	2.58	3.429 (4)	147
C15—H15A···Cg1^ii^	0.96	2.97	3.590 (3)	124

Symmetry codes: (i) *x*−1/2, −*y*+1/2, *z*−1/2; (ii) −*x*, −*y*, −*z*+1.
